# Real-Time PCR-Coupled CE-SELEX for DNA Aptamer Selection

**DOI:** 10.5402/2012/939083

**Published:** 2012-08-08

**Authors:** Patrick Ruff, Rekha B. Pai, Francesca Storici

**Affiliations:** School of Biology, Georgia Institute of Technology, 310 Ferst Drive, Atlanta, GA 30332, USA

## Abstract

Aptamers are short nucleic acid or peptide sequences capable of binding to a target molecule with high specificity and affinity. Also known as “artificial antibodies,” aptamers provide many advantages over antibodies. One of the major hurdles to aptamer isolation is the initial time and effort needed for selection. The systematic evolution of ligands by exponential enrichment (SELEX) is the traditional procedure for generating aptamers, but this process is lengthy and requires a large quantity of target and starting aptamer library. A relatively new procedure for generating aptamers using capillary electrophoresis (CE), known as CE-SELEX, is faster and more efficient than SELEX but requires laser-induced fluorescence (LIF) to detect the aptamer-target complexes. Here, we implemented an alternative system without LIF using real-time- (RT-) PCR to indirectly measure aptamer-target complexes. In three rounds of selection, as opposed to ten or more rounds common in SELEX protocols, a specific aptamer for bovine serum albumin (BSA) was obtained. The specificity of the aptamer to BSA was confirmed by electrophoretic mobility shift assays (EMSAs), an unlabeled competitor assay, and by a supershift assay. The system used here provides a cost effective and a highly efficient means of generating aptamers.

## 1. Introduction

Aptamers are short single-stranded oligomers made up of DNA, RNA, or peptides that are capable of binding a target ligand (proteins, small molecules, or even living cells) with high affinity. They are also known as artificial antibodies because in addition to binding with high affinity, they also bind with high specificity. Aptamers have several advantages over antibodies, including ease and low cost of production which does not involve animals. Aptamers are less immunogenic than antibodies and are already being used as therapeutic agents in humans [1]. Nucleic acid aptamers are also able to act in ways that antibodies cannot. Nucleic acid aptamers, unlike antibodies, can be selected for and used under nonphysiological conditions, such as high-salt conditions and varying pH [2]. Also, nucleic acid aptamers are able to undergo specific conformational changes that antibodies cannot. For example, nucleic acid aptamer binding can be “turned off” by the addition of the complementary strand [3]. Additionally, nucleic acid aptamers can undergo a conformational change when binding to their target and can be used as molecular beacons, fluorescently “off” when unbound and “on” when bound [4]. The field of aptamers is rapidly growing as is the number of applications for their use. 

Nucleic acid aptamers are “evolved” from random sequences of DNA/RNA by a process known as systematic evolution of ligands by exponential enrichment (SELEX) [5]. The SELEX procedure involves the use of the random library of DNA/RNA sequences being incubated with the target, followed by a partitioning step to remove unbound sequences, an elution step to recover the binding sequences, and then an amplification step to generate a library of sequences enriched for binding. The SELEX procedure generally takes months to complete, with a typical selection requiring 10 or more rounds before completion [6]. Also, traditional SELEX requires a support for the target (magnetic beads, membranes, etc.) to bind with. The supports themselves can be targets for selection, and often rounds of negative selection must be done to avoid aptamers for the support. 

Use of capillary electrophoresis (CE) allows for SELEX to be performed in a much shorter amount of time due to much more efficient partitioning and without the aptamers binding to the ligand support (the ligand flows freely in buffer, there is no support). In as little as one round of selection [7], and generally less than five rounds of selection, strong binding highly specific aptamers may be obtained. CE-SELEX is a new technology first developed for use in 2004 and has yet to be commonly used [8]. One of the main advantages to CE-SELEX over traditional SELEX is that the aptamer-target complex can be detected in the first round of selection. This early detection contrasts traditional SELEX, where several rounds must be done before being able to detect any DNA [9]. Most CE-SELEX is done with laser-induced fluorescence (LIF) to increase the detection sensitivity to the analyzed samples. Using CE with LIF, a laser excites fluorescently labeled samples passing through the glass capillary tube which then emits light that is captured by an on-board detector attached to the CE machine itself. 

We have developed a technique for selection of DNA aptamers using CE but without the need for an on-board laser/detector system. The system takes advantage of real-time polymerase chain reaction (RT-PCR). RT-PCR is able to sensitively detect DNA-target complexes early in the selection procedure with an efficacy greater than that of traditional SELEX and equal to that of CE-SELEX with LIF. We believe that this system could be beneficial for researchers that have access to CE, but do not have access to CE with LIF, and are seeking to perform CE-SELEX. The protein that we chose to use as a target for aptamer selection was bovine serum albumin (BSA).

BSA is one of the most commonly used proteins in biochemical studies. BSA is widely used for stabilization of enzymes, preventing enzymes of interest from adhering to tubes or pipettes, or as a protein comparison standard. There are several advantages to using BSA in that it is relatively stable, abundantly available from cow's blood, and is of low cost. Due to the low cost and high abundance of BSA, we chose it to serve as the protein target for our RT-PCR-coupled selection system. Here, we report the isolation of a DNA aptamer with specificity for BSA using the RT-PCR coupled capillary electrophoresis selection system.

## 2. Materials and Methods

### 2.1. Aptamer Selection

The target protein of interest, bovine serum albumin (BSA), was purchased as a lyophilized powder through Sigma-Aldrich and was greater than 98% pure. The BSA was dissolved in RB1 buffer (50 mM Tris-HCl, pH 8.2) and stored at 4°C at a stock concentration of 1 mM. The RB1 buffer was also used as the run buffer for capillary electrophoresis. The DNA library was purchased from Alpha DNA and contained a sequence of 5′_CTTCTGCCCGCCTCCTTCC-(N)36-GACGAGATAGGCGGACACT_3′ (36 random nucleotides flanked by two-fixed 19 base regions used later as primers for PCR amplification).

The protocol for SELEX using capillary electrophoresis (CE) was essentially as described earlier [10] but with a few modifications. The initial bulk affinity assay was performed with 50 *μ*M BSA and 25 *μ*M DNA in order to view any DNA-protein complexes and determine the collection window. Capillary electrophoresis was done using a Beckman Coulter ProteomeLab PA 800 with a photon diode array (PDA) capable of reading wavelengths in the UV range (10 nm–400 nm). Separation was done at a voltage of 10 kV and with 0 psi of pressure, except that during timed fraction collection the machine automatically reaches 0.1 psi of pressure. After determination of the collection window based on the bulk affinity analysis, the first round of selection began. The initial *in vitro* selection procedure involved 500 nM BSA and 3.3 *μ*M DNA. The DNA library (0.3 *μ*L at 100 *μ*M) was mixed with 0.3 *μ*L of SB3 (100 mM Tris-HCl at pH 8.2, 200 mM NaCl, and 10 mM MgCl_2_) for a final concentration of 50 *μ*M DNA library, 50 mM Tris-HCl at pH 8.2, 100 mM NaCl, and 5 mM MgCl_2_. This mixture was heated in the BioRad iCycler to 94°C for 1 minute and then cooled to 20°C at a rate of 0.5°C per second. After the folding of the DNA library, 5 *μ*L of 1 *μ*M BSA dissolved in RB1 buffer was added, and additional run buffer was added to make the final volume 10 *μ*L. This brought the final concentrations to 3.3 *μ*M DNA library, 500 nM BSA, 6 mM NaCl, 300 *μ*M MgCl_2_, and 50 mM Tris-HCl (pH 8.2). The collection window was partitioned into seven different fractions. 

### 2.2. RT-PCR

After each round of selection, fractions were analyzed through real-time PCR (RT-PCR) using the ABI StepOne Plus. RT-PCR was done with two primers: the forward aptamer-amplifying primer P1 (5′_CTTCTGCCCGCCTCCTTCC_3′) and the reverse primer P2 (5′_AGTGTCCGCCTATCTCGTC_3′), respectively. The primers were designed using OligoAnalyzer (http://www.idtdna.com/analyzer/Applications/OligoAnalyzer/) to limit complementarity to each other, in order to decrease nonspecific amplification of self-dimerizing primers. For amplification, 20 *μ*L of PCR mix was prepared consisting of 10 *μ*L of 2X Quanta SYBR Green PCR Master Mix (Roche), 0.6 *μ*L of 10 *μ*M P1, 0.6 *μ*L of 10 *μ*M P2, 1 *μ*L of collected fraction as template, and 7.8 *μ*L H_2_O. The PCR setup was as shown in Table 1.

### 2.3. Amplification and Isolation of Aptamer Strand

Following RT-PCR, the fraction containing the complex was amplified using standard PCR. PCR was done in a 100 *μ*L volume consisting of 1 *μ*L of 5 U/*μ*L Ex Taq polymerase from Takara, 3 *μ*L of 10 *μ*M forward primer P1, 3 *μ*L of 10 *μ*M reverse primer P2, 10 *μ*L of 10X Mg^2+^ buffer (Takara Ex Taq), 8 *μ*L of 2.5 mM each dNTP, and 5 *μ*L of the collected fraction from capillary electrophoresis. PCR was done using primer P1 and P2 as noted previously, except that the number of cycles for PCR amplification was based on 50% of the maximum yield as determined by RT-PCR. Additionally, primer P2 was biotinylated at its 5′ end. The biotin-labeled primer was used subsequent to PCR in order to separate the strand of interest and the nonaptamer strand after PCR. Magnetic beads with streptavidin coating from Bangs Laboratories (Biomag Nuclease-Free Streptavidin) were used to bind the biotin-labeled DNA. Strands were separated with 10 mM NaOH after three washes with wash buffer (10 mM Tris-HCl at pH 8 with 500 mM NaCl and 1 mM EDTA). The single-stranded aptamer pool was used in subsequent rounds of selection.

### 2.4. Cloning and Sequencing

Post-selection DNA cloning of the aptamer pool was done with the Zero Blunt TOPO PCR Cloning Kit (Invitrogen). Standard PCR with unlabeled primers P1 and P2 was used to generate double-stranded DNA containing the aptamer sequence, which was then blunt-end ligated into the pCR-Blunt II-TOPO vector that contains the kanamycin resistance gene. Colonies were selected for growth on kanamycin-containing media (kanamycin final concentration was 40 *μ*g/mL), and plasmid DNA was isolated using the GeneJET Plasmid Miniprep Kit (Fermentas). Asymmetric PCR with unlabeled primers P1 and P2 was used on the plasmid DNA to predominately generate the strand of interest which was then analyzed using CE and RT-PCR. Twelve colonies were pooled into a group and each group was assayed for BSA binding using CE. Individual plasmids from each pooled group that showed binding were sequenced by Eurofins MWG Operon. Based on sequencing results, several candidate aptamers were chosen and ordered as salt-free oligonucleotides. Consensus sequence was analyzed using ClustalW2 (http://www.ebi.ac.uk/Tools/msa/clustalw2/). Additionally, mFold (http://mfold.rna.albany.edu/?q=mfold/DNA-Folding-Form) was used on each candidate aptamer to identify secondary structure.

### 2.5. Electrophoretic Mobility Shift Assay (EMSA)

Potential aptamer oligonucleotides and a negative control oligonucleotide were 5′ labeled with P32 *γ*-ATP using T4 Polynucleotide Kinase (New England Biolabs). The negative control consisted of an oligonucleotide of the same length as the random DNA library oligonucleotides (74 bases), contained the same flanking primer regions, and had a fixed sequence for its internal region 5′-CTTCTGCCCGCCTCCTTCCGGTCGGGCACACCTGTCATACCCAATCTCGAGGCCAGACGAGATAGGCGGACACT-3′. The internal region was chosen using a random DNA sequence generator with a specified GC content of 50% (http://www.faculty.ucr.edu/~mmaduro/random.htm). EMSAs were performed under different binding conditions. The binding conditions for the initial EMSA were done by adding equal amounts of SB3 buffer to the labeled oligonucleotides (20,000 cpm equivalent) and incubating at 94°C for 1 minute in a PCR machine and a gradual cooling to 20°C at a rate of 0.5°C per second (total time taken is ~4 minutes). BSA stock of 1 mM was made in RB1 buffer (the run buffer used in the CE-SELEX protocol). Different dilutions of BSA, as required for assessing aptamer binding, were also made in RB1 buffer. With fixed concentration of the *γ*P32-labeled candidate aptamer, the concentration of the ligand (BSA) was varied from 50 to 800 *μ*M.

 The buffer used for binding was a 6X buffer consisting of 600 mM ammonium chloride, 300 mM potassium chloride, 30 mM sodium chloride, 120 mM Tris-HCl pH 7.5, 30% glycerol, and bromophenol blue (BPB) 0.25%. The entire binding conditions were made up to 10 *μ*L with RB1 buffer. The reaction was incubated at room temperature for 30 minutes after which 1.5 *μ*L of 10X loading buffer (200 mM Tris-HCl pH 8.2, 50% glycerol, and BPB 0.25%) was added and the reaction was left on ice for 5–15 minutes before loading into the gel. The reaction complex was run on an 8% polyacrylamide gel under nondenaturing conditions. Minigels were made with stock solutions of 40% acrylamide/bis-acrylamide (29 : 1), 1X Tris-borate EDTA (TBE), 10% ammonium persulfate (APS), and tetramethylethylenediamine (TEMED). Gels were run using the Mini-PROTEAN Tetra Cell apparatus from BioRad. Prerun was done in 1X TBE buffer for 1 hour prior to loading of the samples. The samples were run at 150 V until the bromophenol blue dye reached the bottom of the gel. The radioactivity in the gel was analyzed by Phosphor Imager (Molecular Dynamics-Typhoon Trio Imager). After the initial EMSA, binding conditions were slightly changed. Apart from the above-mentioned 6X buffer, an additional 5X binding buffer containing 100 mM Tris-HCl pH 8.5, 250 mM NaCl, 10 mM MgCl_2_, 10 mM ZnCl_2_, and 10% glycerol was added in the incubation mixture. Furthermore, the radiolabeled oligonucleotides were added directly to the incubation mixture and not heated to 94°C as done previously. samples were left on ice for 5–15 minutes before being loaded into the 4% nondenaturing gels which had been prerun for 1 hour in 1X TBE as described previously.

### 2.6. Competition Assays

Increasing concentrations of the specific unlabeled oligonucleotide were added during incubation of the BSA-aptamer complex (conditions described previously). Three different amounts (1.25, 2.5, and 5 pmol) were used. Complex formation was allowed to go on for 30 minutes at room temperature before the radioactively labeled oligonucleotide was added and the incubation was continued for another hour and 30 minutes. In the control sample, all the conditions were similar except for the absence of the unlabeled competing oligonucleotide. 

### 2.7. Supershift Assays to Determine Specificity of Binding of BSA to the Potential Aptamer

EMSAs were set up using 4 *μ*g of the anti-BSA polyclonal antibody (obtained from Invitrogen). Essentially, the antibody (4 *μ*g) was mixed with BSA (500 *μ*M) along with the 5X binding buffer and left on ice for 1 hour. The radioactively labeled I1-5 aptamer and the 6X buffer were added next and the incubation continued for 1 hour and 30 minutes at room temperature before stopping the incubation by leaving the samples on ice for 5–15 minutes. Next, the EMSA proceeded as previously described.

## 3. Results and Discussion

### 3.1. CE Machine Calibration and Initial Runs

**Figure 1:**
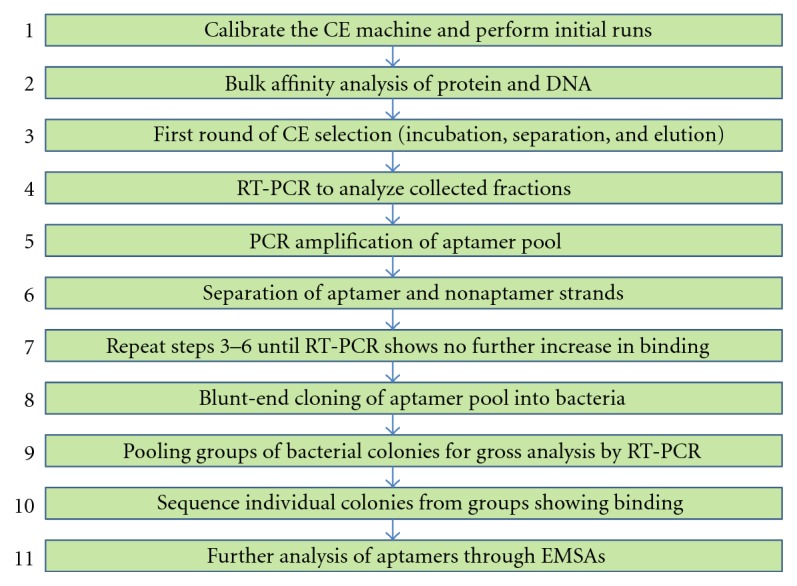
Summary of RT-coupled CE-SELEX. The overall scheme of the protocol for selection is laid out sequentially. The scheme omits the steps after selection of the aptamers. Further analysis may be carried out to confirm the specificity and affinity of the aptamers, but this is specific to the user and separate from the selection.

The general scheme of RT-PCR coupled CE-SELEX is shown in Figure 1. To test the concept that aptamer selection with CE-SELEX and RT-PCR could be feasible, we first began by calibrating the capillary electrophoresis (CE) machine. The CE machine used, the ProteomeLab PA 800, is incapable of running solutions that contain high concentrations of salts. Due to this limitation, our buffers had to be tested to find the proper salt balance to ensure that the machine did not fail but at the same time had enough salt to stabilize our aptamer structures. Therefore, the buffer condition used for the initial step of folding the DNA library was done in a high-salt buffer (50 mM Tris-HCl at pH 8.2, 100 mM NaCl, and 5 mM MgCl_2_) followed by incubation and running the folded DNA library in low-salt concentrations (50 mM Tris-HCl at pH 8.2, 6 mM NaCl, and 300 nM MgCl_2_) to ensure both proper folding and running of the DNA library. Although the low-salt problem may have excluded many potential binders to BSA, low-salt concentrations actually mirror the physiological conditions more accurately since the cellular level of magnesium is only ~1-2 mM [11]. After optimizing for the salt concentrations, we used CE and ran a large amount (100 *μ*M) of BSA alone in order to visualize the free protein peak run timing. Next, we performed the same analysis using a large (3 *μ*M) amount of DNA alone. 

### 3.2. Bulk Affinity Analysis and Selection

**Figure 2:**
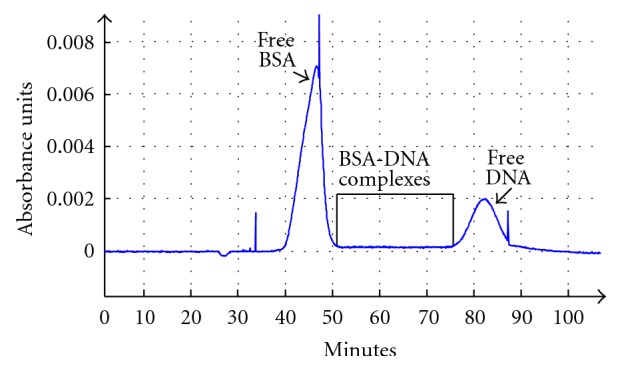
Bulk affinity assay of BSA and DNA. This electropherogram is the bulk affinity assay in which a high concentration of BSA (100 *μ*M) and DNA (3 *μ*M) was run together. The *y*-axis represents the absorbance at 280 nm, and the *x*-axis is the run time to the absorbance detector. The tall peak starting around 40 minutes is the free BSA, and the smaller peak starting around 77 minutes is the free DNA. The collection window for the BSA-DNA complexes is boxed in black.

**Figure 3:**
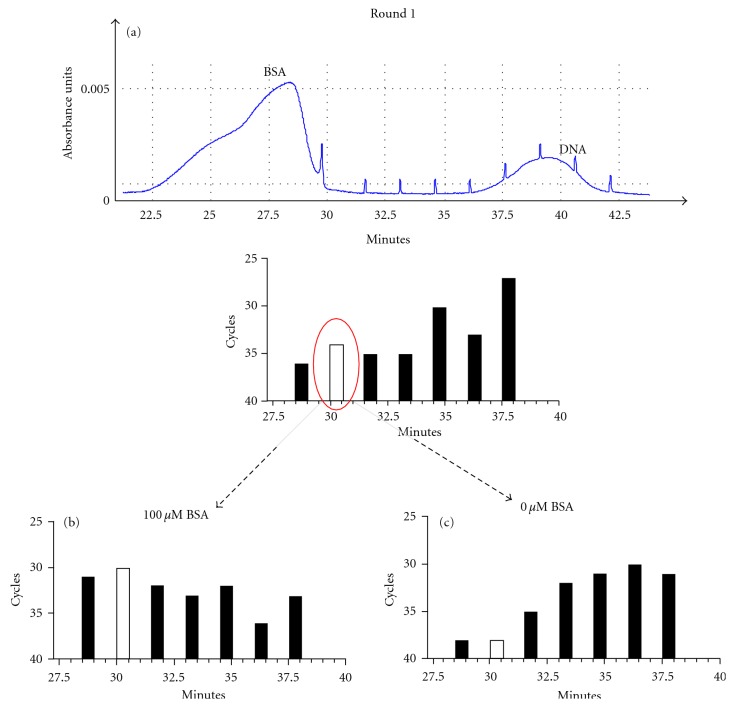
CE fractions collected and analyzed. (a) The time points at which the seven fractions were collected during the first round of CE selection are represented by the bars in the graph. The *x*-axis for the bar graph and the electropherogram is the run time to the absorbance detector in minutes. The *y*-axis of the electropherogram represents the absorbance at 280 nm. The *y*-axes for the bar graphs are the number of RT-PCR cycles it took to reach 50% of the maximum or the midpoint cycle. The earlier the fraction reached the midpoint, the more DNA the fraction contained. The electropherogram above the bar graph is shown as a guide since the actual amount of BSA used for round 1 of selection is very low and it cannot be detected by CE. A supposed complex was detected in the second fraction collected during round 1 of selection (white bar). The supposed complex in the 2nd fraction of round 1 was analyzed in two test rounds using 100 *μ*M BSA (b) or no BSA (c). Seven fractions were collected at the same time points as the seven fractions shown in (a) and the corresponding RT-PCR bar graphs of the midpoints are shown, with the 2nd fraction collected shown in white.

Once the individual free protein and free DNA run times were established, we proceeded to combine BSA and DNA for a bulk affinity analysis (Figure 2). The bulk affinity analysis allowed us to visualize peaks for the free BSA and the free DNA in combination, and this information was used to determine the collection window. The CE machine used was the ProteomeLab PA 800 from Beckman Coulter, which did not have laser-induced fluorescence (LIF). To sensitively detect protein-DNA complexes between the free DNA and free protein, we relied on RT-PCR. The collection window started from the end of the free-protein peak to the start of the free-DNA peak (Figure 3(a)). This collection window was further subdivided into seven separate two-minute fractions. The seven fractions, instead of just one fraction, allowed us to analyze with greater precision the region of the DNA-protein complex. Unlike the bulk affinity assay in which 100 *μ*M BSA was used, for the selection, the concentration of BSA was reduced to 500 nM in order to increase the stringency of selection. In the first round of selection, the initial collected fraction of DNA detected by RT-PCR needed 36 cycles of amplification to reach 50% of the maximum yield, the midpoint cycle, of PCR (Figure 3(a)). The second fraction analyzed had a higher amount of DNA compared to the first fraction collected, taking only 34 cycles of amplification to reach the midpoint cycle. The amount of DNA collected for the third and fourth fractions was lower than that of the second fraction collected. The fifth fraction collected showed a marked increase in DNA indicative of the beginning of the free-DNA peak and so this fraction was not used. The pattern detected by RT-PCR implied a DNA-protein complex present in the second fraction collected since this fraction was collected by CE after the free protein peak, but well before the free-DNA peak.

 The collected DNA containing the supposed DNA-protein complex was RT-PCR amplified using primers flanking the internal random sequence. The forward primer, P1, amplified the aptamer-containing strand of interest, while the reverse primer, P2, amplified the nonaptamer complementary strand. In a previous protocol to select DNA aptamers, it was shown that overamplification of the random library leads to formation of nonspecific products [12], therefore the random library was only amplified to ~50% of the maximum yield as measured by RT-PCR. After RT-PCR analysis revealed the optimum number of cycles to amplify the collected DNA, a regular PCR using more of the collected DNA was performed. In this regular PCR, the P2 primer was labeled at its 5′ end with biotin while P1 had no modifications. After the PCR, the DNA products were attached to streptavidin-coated beads due to the strong interaction between the streptavidin on the beads and the biotin-labeled primer P2. After immobilization on the beads and several washing steps, the forward aptamer-containing strand was released from the complementary strand by incubation with 10 mM NaOH. After this step, the binding of the new aptamer pool was analyzed using CE and RT-PCR. 

To assess the binding of the newly generated aptamer pool to the target, two separate CE runs were done, one with a 200-fold molar excess of BSA used for the first round of selection (100 *μ*M) (Figure 3(b)) and another without any BSA (Figure 3(c)). Based upon these CE test runs, it was clear that the second fraction collected in round one of CE-SELEX did show binding to BSA. The aptamer pool (5 *μ*L of the collected fraction) was then subjected to another round of selection. Selection continued until the fourth round; at which point RT-PCR did not show any change in the amount of DNA at the complex and we did not continue with any further rounds of selection.

### 3.3. Postselection Characterization of Potential Aptamers

 Potential aptamers collected during the third round of selection were again amplified and then used for cloning. Instead of amplifying with a biotin-labeled P2 primer, the potential aptamers were amplified with a nonlabeled P2 primer since the biotin label at the 5′ end of the DNA might interfere with ligation during cloning. TOPO blunt-end cloning was performed with the aptamer pool and colonies were selected by growth on LB plus kanamycin plates. Two hundred colonies were selected and analyzed for BSA binding using CE and RT-PCR. Groups of 12 colonies were combined in order to facilitate faster analysis. In order to amplify the aptamer strand from the vector with the aptamer insert, asymmetric PCR was performed using a 10-fold excess of the aptamer strand primer. Four groups: I1, R2, G2, and O3 showed stronger binding than the others by RT-PCR and then the individual clones from these groups were sequenced. 

After sequencing, in order to confirm that the potential aptamer sequences were binding to BSA, salt-free oligonucleotides containing the potential aptamer sequences were used in EMSA gels. EMSAs work on the principle that DNA bound in a DNA-target complex will run slower than free DNA. The free DNA will appear at the bottom of the gel, while the DNA bound in the DNA-target complex will be shifted higher up in the gel. The potential aptamers, as well as a negative control (described in Section 1), were initially screened with a set of binding conditions similar to that of the CE experiments. Only those sequences that were 76 or 75 bases in length, and identical or almost identical in length to the original library length of 76 bases, were ordered and subsequently screened by EMSA. The candidate aptamer sequences were analyzed *in silico* by using ClustalW2 and mFold, but no consensus sequence or specific secondary structure between the sequences was found.

**Figure 4:**
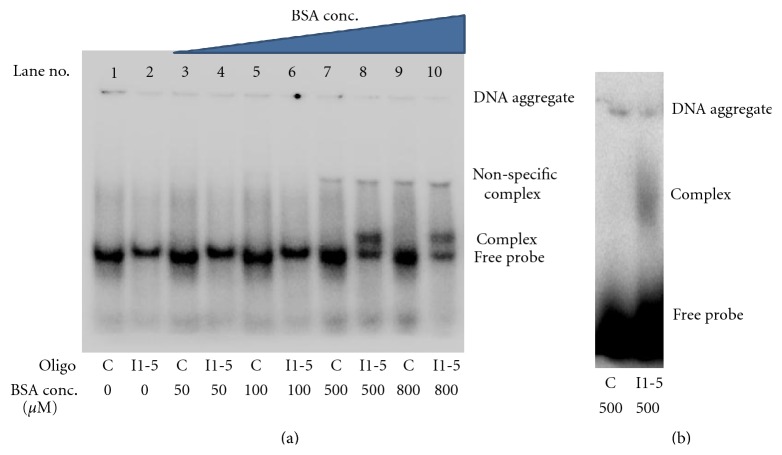
Confirmation of CE-SELEX aptamer selection by EMSA. (a) Shown is the initial EMSA done with the BSA aptamer, I1-5, selected through RT-PCR coupled CE-SELEX, along with a negative control of the same length (“C” in the figure) described in Section 2. At 500 *μ*M BSA a specific complex, formed between the BSA and DNA. Also, there is a higher band corresponding to nonspecific interaction at high concentrations of BSA in both the negative control and the aptamer I1-5 sequences. (b) EMSA of I1-5 and negative control with the modified binding conditions. The binding pattern was the same with these two different binding conditions and for clarity only the BSA at 500 *μ*M is shown.

**Figure 5:**
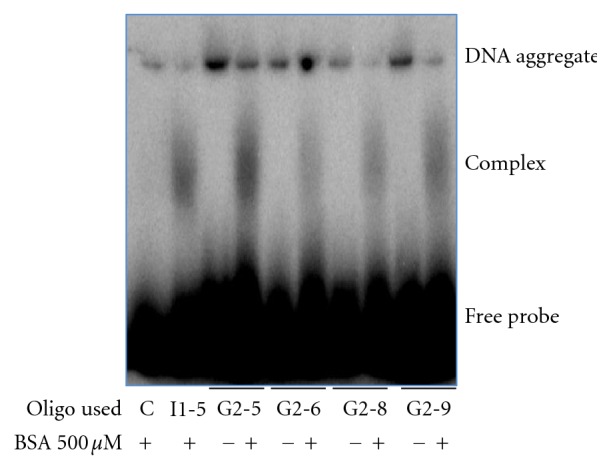
EMSA gel confirming binding of group G2 aptamers. Shown is a screen done with potential aptamers from the second group tested, G2. Several from this group were capable of binding to BSA. I1-5 was used as a positive control for binding, and the negative control (“C”) was also used.

Of the initial group of oligonucleotides screened by EMSA, the I1 group, one member of this group of potential aptamers, I1-5, did bind to BSA and a distinct bound complex could be visualized at 500 and 800 *μ*M BSA in the initial EMSA (Figure 4(a)). The bound complex showed a modest, but distinct shift. Also in the initial EMSA, at higher concentrations of BSA (>500 *μ*M), a faint band could be seen. This band was considered a nonspecific complex because both the negative control and the I1-5 aptamer had this band. In this EMSA, and in all subsequent EMSAs, the appearance of radiolabeled DNA at the top of each lane, in the well, can be seen. The DNA at the top of the lanes is caused due to the single-stranded nature of the DNA. The single-stranded DNA is capable of forming aggregates with itself and with the loading dye. The size of these aggregates prevents the DNA from entering the gel and thus it remains in the well. After the initial EMSA modeled after the CE experiments, binding conditions were changed to include higher salt conditions that were not possible to run with the ProteomeLab PA 800. These higher salt conditions were used to stabilize the aptamer secondary structure. In addition to buffer conditions, we also did not subject the aptamers to the heating at 94°C prior to incubation. Again the potential aptamer I1-5 showed a bound complex using these modified binding conditions (Figure 4(b)). After the initial screen for potential aptamers with the I1 group, a subsequent screen using oligos from the G2 group and the modified EMSA conditions revealed several additional aptamers for BSA (Figure 5). The list of the potential aptamers' sequences ordered is shown in Table 2, among these aptamers, I1-5 was chosen for further characterization.

### 3.4. Further Characterization of I1-5 Aptamer

**Figure 6:**
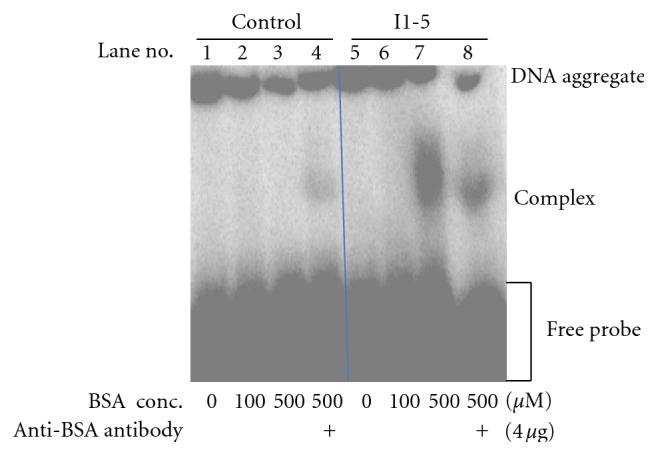
Supershift assay with inclusion of antibody to BSA binding in gel retardation assays. A polyclonal antibody to BSA was tested for binding to BSA in the reaction complex and analyzed by EMSA. The negative control previously shown not to bind to BSA was used as well as I1-5, the BSA aptamer. For the negative control, no binding to BSA was seen at 100 *μ*M or 500 *μ*M BSA (lane 2 and 3). With addition of 4 *μ*g of the antibody, there was some nonspecific binding, as seen by the faint band, by the control sequence (lane 4). However, with I1-5 there was binding to 500 *μ*M BSA (lane 7), but this binding was disrupted when the antibody was incubated with BSA prior to the EMSA (lane 8). As stated previously, some of the radiolabeled DNA formed aggregates incapable of entering the gel, giving rise to the bands at the top of the lanes. A slight leak occurred during gelling such that the top of lane 8 appears disjointed due to the shape of the gel.

**Figure 7:**
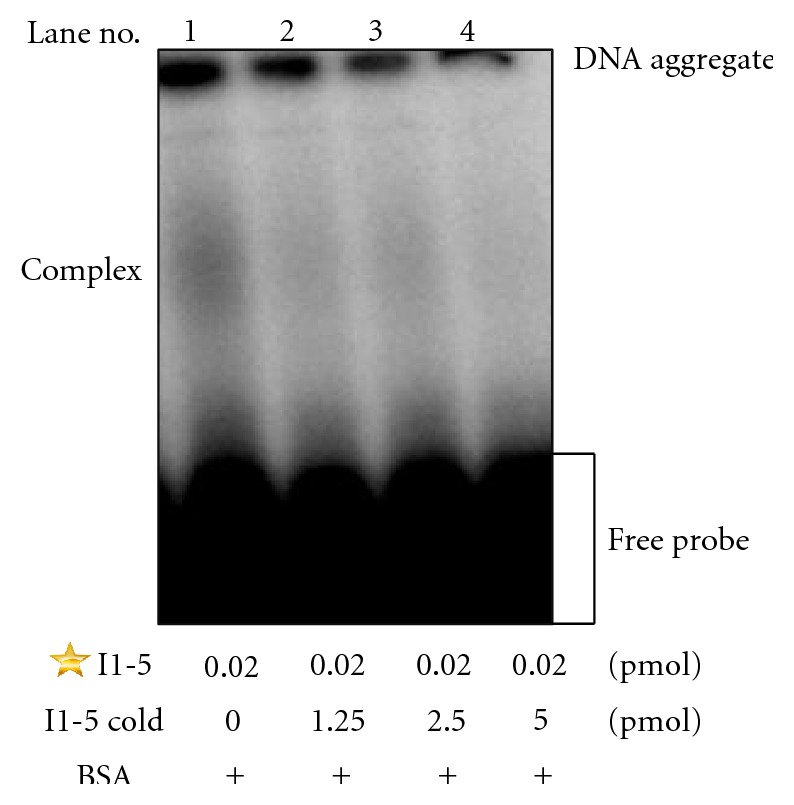
Competition assay with unlabeled I1-5 aptamer. Unlabeled “cold” I1-5 aptamer at 0, 1.25, 2.5, and 5 pmol as indicated were incubated with 500 *μ*M BSA (indicated by the “+”) before addition of the P32-labeled I1-5 (represented by a star) aptamer. Again, the formation of DNA aggregates is present at the top of the lanes. As unlabeled DNA competitor increases, the amount of radiolabeled DNA trapped in the aggregates decreases as expected.

Only the I1-5 oligonucleotide was further pursued as it was the first aptamer discovered by the screen that consistently showed binding ability to BSA in the CE-SELEX as well as under the two different EMSA conditions. To further confirm the specificity of this aptamer to BSA, a supershift assay using an antibody to BSA was performed. In the presence of 4 *μ*g of the anti-BSA polyclonal antibody, the binding capacity was reduced as seen by an appreciable reduction in the intensity of the radioactive oligo banding pattern (Figure 6). This supports the idea that the anti-BSA polyclonal antibody and the BSA aptamer I1-5 recognize the same epitope. A similar pattern of banding was reported for the human neurofilament mRNA binding to superoxide dismutase1 (SOD1) in a supershift assay [13]. Also, competition assays were performed by incubating BSA with an excess of unlabeled I1-5 aptamer of three different amounts (1.25, 2.5, and 5 pmoles) before the addition of the labeled I1-5 oligo. The BSA-aptamer bound complex was abolished in the presence of higher amounts of the unlabeled competitor (Figure 7). Although by EMSA the I1-5 aptamer showed low binding affinity to BSA, the cold-competitor assay, as well as the supershift assay indicated that a specific interaction was occurring between BSA and the I1-5 aptamer, thus supporting the effectiveness of RT-PCR coupled CE-SELEX.

## 4. Conclusion

The conclusion drawn from our studies is that we have been able to generate an aptamer for BSA with appreciable specificity in a few rounds of selection from a random library of DNA oligonucleotides. 

The protocol described here is very efficient compared to the traditional SELEX which takes much more time and reagents. As noted previously, traditional SELEX makes use of a solid support to which the target must be bound. The coupling of the target to the support is not completely efficient and often much of the target remains unbound and washed away. Using CE-SELEX, there is no need for a solid support and because of the small size of the capillary a much smaller amount of target is required. Even though CE-SELEX is much more efficient than traditional SELEX, CE-SELEX still requires additional equipment (LIF) beyond the standard capillary electrophoresis machine. CE is not restricted to the isolation of aptamers, and while many labs may have a CE machine, much fewer will have one with LIF. Our system can be used with any CE machine, with or without LIF.

Another advantage of the system is the high level of sensitivity. RT-PCR and LIF both work under the principle of fluorescence. Very sensitively and directly, RT-PCR has the potential to be more sensitive because unlike LIF, which directly detects the fluorescent signal, RT-PCR can amplify the signal. By amplifying a signal undetectable by LIF, DNA-target complexes that would have been missed by LIF could be detected and collected using our system. Taken together the efficiency, time, and sensitivity of the protocol for DNA aptamer selection by coupling CE-SELEX with RT-PCR described here can benefit other researchers that are also interested in selecting for aptamers and would like to use CE-SELEX, but are unable to do so for lack of LIF.

 BSA was chosen to test our RT-PCR-coupled selection system, but the BSA aptamer we selected may be beneficial itself. Due to the high amount of BSA used in many biochemical assays, sometimes the amount of BSA present in a mixture of proteins obscures accurate readings of proteins of interest. It would be useful to have a way to deplete this unwanted BSA from a reaction. Also, BSA can be allergenic [14] and in certain situations it might be beneficial to detect small amounts of BSA contaminant present in cow's milk. Currently, antibodies to BSA provide an answer to these issues, but as previously mentioned, there are several advantages to using aptamers over antibodies.

## Figures and Tables

**Table 1:** 

Cycle no.	Denaturation	Annealing	Extension
1	94°C for 30 seconds		
2–50	94°C for 10 seconds	55°C for 10 seconds	72°C for 10 seconds
51			72°C for 1 minute
Hold at 4°C			

**Table 2:** Sequences of potential aptamers for BSA. The table contains the sequences of the potential aptamers from each of the pooled groups of clones that showed binding to BSA by RT-PCR analysis. These sequences were ordered as salt-free oligonucleotides and analyzed using electrophoretic mobility shift assays (EMSAs), and sequences that showed binding are bolded. I1-5, boxed in black, was chosen for further analysis.

Potential aptamer	Internal sequence
O3-9	CAGTCACCTCAGCGTCCACAATTATGGCAGCGCGCG
G2-1	TTTATTGGGGGAATATAACCGATCATGTGGCGGTTA
G2-2	ATACAATGATTTGCACACCGGGTGGCGTGAATTGT
G2-3	GGATTGTGGTAAGCTCTATCTACCTTGCTACGCCC
G2-4	GGCTGATCGACAATAAGCGTGCCGGCCCTCAACTCC
**G2-5**	**TCCGATGCCACACTTGACGTGCTGGGTTGGCTGCGT**
**G2-6**	**AGCGAGAGGCGACTTCATTATTGGGAGGTGGCTTCG**
**G2-8**	**AGTAGGTGTTATGAGCACGACCGGTTTACTTCACG**
**G2-9**	**GGAGTGACTGGAGGATCTGGTCCCCGGAGTTCCTTG**
G2-11	GCATGTGGTGCTTCTTGACCGCCTGCGCCTGCGGGT
R2-6	AACGACTGGACTCCGTCATACAGTTTGGGGAAAGG
R2-8	TCCCTACCCGGTGTTTGAAACGGCCGTAAGGAACTT
R2-10	GATAGTAAGACCTGATTGGGGTCAAGCTAACGTCGA
R2-11	GCTCGCGTTGTAAGCATTTGTTGGNCGAGATAGGCG

**I1-5**	**GCCCGCCGTGGCTGGGTCTTCCTTGGTCGGTCTAC**

I1-11	GTATCTACAGGGTGGCCAGGGCGCAACCGGGCGAGT
I1-12	TGGCTAGCGATGTATTCCGTTTTGGGGATAAATCCA
